# Performance and Mechanism of Alkylimidazolium Ionic Liquids as Corrosion Inhibitors for Copper in Sulfuric Acid Solution

**DOI:** 10.3390/molecules26164910

**Published:** 2021-08-13

**Authors:** Guocai Tian, Kaitao Yuan

**Affiliations:** State Key Laboratory of Complex Non-Ferrous Metal Resource Clean Utilization, Faculty of Metallurgical and Energy Engineering, Kunming University of Science and Technology, Kunming 650093, China; kaitaoyuan@163.com

**Keywords:** corrosion inhibitor, adsorption, 1-alkyl-3-methylimidazolium hydrogen sulfate, inhibition mechanism, quantum chemical calculations, QSAR, molecular dynamics simulations

## Abstract

The addition of corrosion inhibitors is an economic and environmental protection method to prevent the corrosion of copper. The adsorption, performance, and mechanism of three 1-alkyl-3-methylimidazolium hydrogen sulfate ([BMIM]HSO_4_, [HMIM]HSO_4_, and [OMIM]HSO_4_) ionic liquids (ILs) on the copper surface in 0.5 M H_2_SO_4_ solutions were studied by quantum chemical calculation, quantitative structure-activity relationship (QSAR), and molecular dynamics simulation. It is found that the main reactive site is located on the imidazolium ring (especially the C2, N4, and N7 groups). When the alkyl chain of the imidazolium ring is increasing, the molecular reactivity of the ILs and the interaction between the ILs inhibitor and copper surface are enhanced. The imidazole ring of the ILs tends to be adsorbed on Cu (111) surface in parallel through physical adsorption. The order of adsorption energy is [Bmim]HSO_4_ < [Hmim]HSO_4_ < [OMIM]HSO_4_, which is in agreement with the experimental order of corrosion efficiency. On the imidazole ring, the interaction between the copper surface and the C atom is greater than that between the copper surface and the N atom. It is found that ILs addition can hinder the diffusion of corrosion particles, reduce the number density of corrosion particles and slow down the corrosion rate. The order of inhibition ability of three ILs is [Bmim]HSO_4_ < [Hmim]HSO_4_ < [OMIM]HSO_4_,which agree well with experimental results. A reliable QSAR correlation between the inhibition corrosion efficiency and molecular reactivity parameters of the ILs was established.

## 1. Introduction

Copper and its alloys are widely utilized as an important material in various industrial applications such as microelectronic, military, and civilian living life owing to its electrical conductivity, good thermal properties, mechanical workability, good availability, cheap, and corrosion resistance properties. However, copper will be corroded unavoidably in acid surroundings and humid environments, greatly limiting its use [1,2,3,4,5,6,7,8,9,10,11,12,13]. The common ways for corrosion prevention are isolation protection, electrochemical protection, and adding inhibitors. One effective and useful approach to protect copper metals against the corrosion problem of acid solutions is adding corrosion inhibitors, which are often ionic liquids because they have many special properties such as better thermal stability, good solubility, high conductivity, wider electrochemical window, and so on [5,7,12,13]. Ionic liquids (ILs) are currently used as fascinating compounds which are salts with liquid formation at room temperature or low temperature (<100 ℃) in intense examination [14].

Recently, some ionic liquids (ILs) based on imidazolium, pyridinium, and benzotriazole have been used as corrosion inhibitors for copper and its alloys in acid solution and achieved good results. [14,15]. Ionic liquids are popularly used as sustainable and green corrosion inhibitors for metal [5,7,12,15,16,17,18]. Many researchers used electrochemical tests and surface investigations to obtain the inhibition efficiency and found that the adsorption process of an inhibitor can be described by Langmuir adsorption [19,20]. However, the inhibition mechanisms and effects have not been clearly understood at all. They found that the most effective inhibitors have π-systems or multiple bonds, and organic compounds containing O, P, N, or S atoms have a good corrosion performance [20]. But it is very difficult to choose the favorite ILs as effective corrosion inhibitors for a specific application since there are about 10^18^ kinds of ionic liquids that can be formed with the present available cations and anions. Fortunately, with the development of computer technology and related theory, in recent years, Savaş et al. further applied the quantum chemical study and molecular dynamics (MD) to explore corrosion inhibition mechanisms and corrosion inhibition performance [21]. The quantitative structure-activity relationship (QSAR) correlates the corrosion inhibition performance with various possible mutual factors and establishes a quantitative prediction model of corrosion inhibition performance, which is very important for screening and designing effective corrosion inhibitors [22].

**Figure 1:**
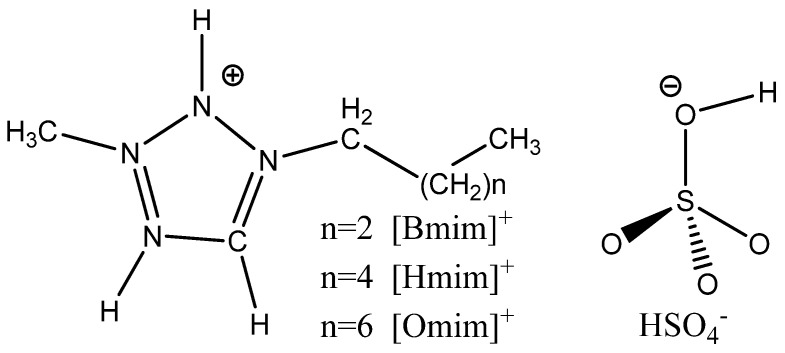
Schematic representation and the atom numbering for the studied ILs.

Three ILs of 1-octyl-3-methylimidazolium hydrogen sulfate ([OMIM]HSO_4_), 1-hexyl-3-methylimidazolium hydrogen sulfate ([HMIM]HSO_4_), and 1-butyl-3-methyl-imidazolium hydrogen sulfate ([BMIM]HSO_4_) (in Figure 1) had been studied experimentally utilized elemental analysis and electrochemical measurement experiment [1]. However, it is not clear how the change of chain length affects the structure and properties of ILs, the interaction between ILs and copper surface, and the adsorption mechanism. This information is very important to understand the mechanism of ILs for copper corrosion inhibition and to develop new inhibitors. Theoretical methods including quantum chemical calculations and molecular dynamics simulations have turned out to be the most appropriate methods for elucidating the inhibitory mechanisms of organic inhibitors [23]. In the present work, a quantum chemical calculation was used to study how the change of chain length affects the structure and reactivity of ILs and their interaction with the copper surface. A molecular dynamics simulation was used to study the adsorption of ILs on a copper surface in vacuum and solution environments and the inhibition mechanism of ILs on corrosion ions in the system. The microscopic molecular active parameters and sites of three ILs inhibitors and adsorption behavior of these three ILs inhibitors on the Cu(111) surface is revealed in detail. Relationships between the microscopic structure parameters and corrosion inhibition efficiency were given with the QSAR method. We showed that theoretical studies can be used as a reliable way to screen green corrosion inhibitors and predict their corrosion efficiency roughly before performing the experiment.

## 2. Results and Discussion

### 2.1. Structure and Reactivity of Three ILs

#### 2.1.1. Optimized Geometry Structures and the Frontier Molecule Orbital Distribution

**Figure 2:**
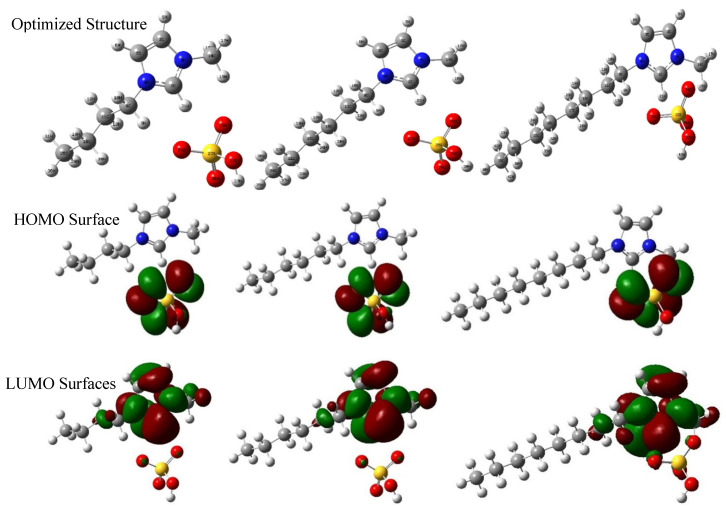
Equilibrium geometry structure, LUMO and HOMO isosurfaces calculated at B3LYP/6-31++G(d,p) for [BMIM]HSO_4_, [HMIM]HSO_4_ and [OMIM]HSO_4_.

The optimized structures of three ILs inhibitors at B3LYP/6-31++G(d,p) are shown in Figure 2. All of the vibrational frequencies are positive, which reflects that the ground optimized structures correspond to global minima. The highest occupied molecular orbital (HOMO) and the lowest unoccupied molecular orbital (LUMO) are also shown in Figure 2. The HOMO level determines the electrons donation ability of a molecule, while the LUMO level determines the electrons acceptation ability of a molecule [24]. From Figure 2, we found that the HOMO surfaces are all localized on the HSO_4_^−^ anion, which means that HSO_4_^−^ can easily provide electrons to metals or other molecules. LUMO surfaces are mainly localized on the imidazolium ring of three ILs, especially the C2 atom, and electron acceptance from the Cu surface in this region is mainly favorable. Because copper can lose electrons in acid conditions, the main reactive site is the imidazole ring of ILs. From the LUMO and HOMO results, it is inferred that the O and S atoms in HSO_4_^−^ anion and the imidazolium ring would be the main reactive site. The increase in chain length increases the distribution of HOMO on the ring and LUMO on the anion, making it easier for ILs to give or accept electrons.

#### 2.1.2. Global Reactivity for Fur ILs in Gas Phase

Table 1 shows the fourteen quantum chemical parameters of three ILs obtained by B3LYP/6-31++G(d,p) in the gas phase. The order of the *E*_HOMO_ is [BMIM]HSO_4_ < [HMIM]HSO_4_ < [OMIM]HSO_4_, which means increasing the alkyl chain can enhance the electrons donation ability of the molecule. The energy gap (Δ*E*) describes the chemical reactivity of a molecule. Molecules with smaller Δ*E* values have higher reactivity from stable adsorption on the metal surface, which is soft and polarizable [21]. The trend of the experimental inhibition efficiency increases with a decreasing value of Δ*E*.

The polarizability is an important indicator. Inhibitors with a high polarizability value will favor accumulation on the metal surface facilitating a strong adsorption process [25]. The trend of increasing experimental inhibition efficiency is consistent with the trend of polarizability. The electrophilicity (*ω*) and electronegativity (*χ*) are useful quantum chemical parameters for predicting the molecular reactivity related to accepting electrons [26]. If the molecule has a lower value of the electronegativity (*χ*) and electrophilicity (*ω*), electrons will be transferred from the high chemical potential of the metal surface to the low chemical potential of a molecule, attracting on the metal surface easily [26]. The order of *χ* and *ω* is [BMIM]HSO_4_ > [HMIM]HSO_4_ > [OMIM]HSO_4_, which is also consistent with the order of experimental inhibition efficiency. Softness (*σ*) and hardness (*η*) are essential molecular properties for analyzing the reactivity and selectivity [27]. A lower *η* value and higher *σ* value represent that the reactivity and selectivity of molecules could be better. The order of *η* is [BMIM]HSO_4_ > [HMIM]HSO_4_ > [OMIM]HSO_4_ and that of *σ* is [BMIM]HSO_4_ < [HMIM]HSO_4_ < [OMIM]HSO_4_, which suggests that adsorption could occur between the molecule and metal surface. Δ*Ν* is equal to the number of electrons transferred between metal and inhibitor. The positive Δ*Ν* follows [BMIM]HSO_4_ < [HMIM]HSO_4_ < [OMIM]HSO_4_, which also confirms that the [OMIM]HSO_4_ has the largest tendency to transfer electrons and tend to interact with the metal surface [28]. Molecular volume (*MV*) represents possible surface coverage on the metal. The molecule with the largest *MV* could provide substantial protection for metal. The tendency of *MV* for three ILs is [BMIM]HSO_4_ < [HMIM]HSO_4_ < [OMIM]HSO_4_.

#### 2.1.3. Local Reactivity

**Figure 3:**
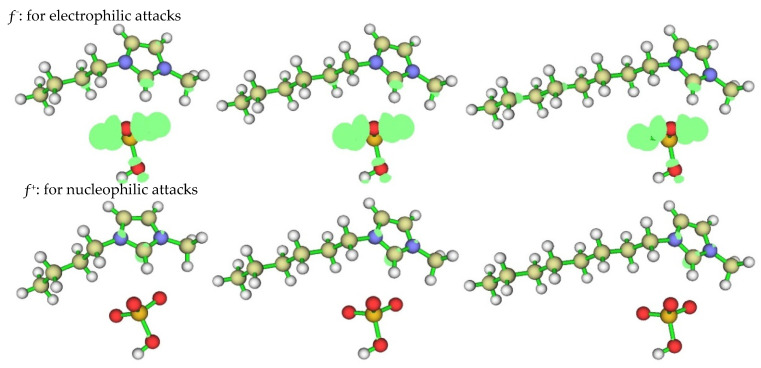
The Fukui function of the nucleophilic attack and electrophilic attack calculated at B3LYP/6-31++G(d,p) for [BMIM]HSO_4_ (**left**), [HMIM]HSO_4_ (**middle**) and [OMIM]HSO_4_ (**right**).

Fukui function *ƒ*(r) is often used to predict local reactivity and confirm the behavior of different sites in a molecule [29]. The preferred sites for nucleophilic attacks and electrophilic attacks represent the region/atom with the highest value of *ƒ*^+^ and *ƒ*^−^ respectively. The electrophilic attack and nucleophilic attack of three ILs obtained by B3LYP/6-31++G(d,p) were given in Figure 3. From Figure 3, we found that the preferred location for electrophilic attacks (*ƒ*^−^) is mainly located on the anion, especially the O and S atoms and C2 of the imidazolium ring. The preferred sites for nucleophilic attack (*ƒ*^+^) are located on the C2, N4, and N7 atoms at the imidazolium ring (see the atomic number of ILs in the stable geometry in Figure 2). The imidazolium ring, the O and S atoms of anion would be the reactive sites, whether for electrophilic attacks or nucleophilic attacks, which agrees with the experimental deduction that the adsorption would have occurred through polar centers as a nitrogen atom in the -C=N- group. Meanwhile, the presence of the electron-donating group on the imidazolium compound structure will increase the electron density on the nitrogen of the -C=N- group [1]. From Figure 3, we found that the increase in chain length of the imidazolium ring increases the distribution of *ƒ*^+^ and *ƒ*^−^, making it easier for ILs to give or accept electrons, which is in agreement with the experimental prediction that the adsorption is more pronounced with an increase in the carbon chain length of the alkyl connecting with the N of imidazolium ring due to their electron-releasing ability. Therefore, compound [OMIM]HSO_4_ is the best inhibitor, and the corrosion inhibition efficiency follows the order: [OMIM]HSO_4_ > [HMIM]HSO_4_ > [BMIM]HSO_4_. Based on the discussion above, it can be concluded that imidazolium molecules, which had a number of active centers (N, O and S atoms), will form a good protective layer on the copper surface to retard its further corrosion.

#### 2.1.4. Electrostatic Potential (ESP) Diagrams

**Figure 4:**
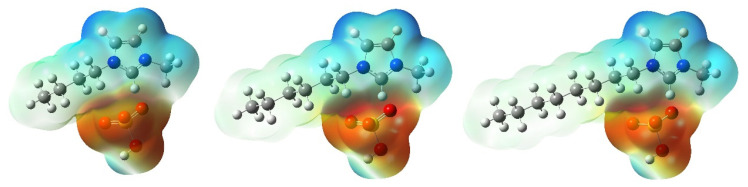
Electrostatic potential (ESP) maps for the studied ILs fromB3LYP/6-31++G(d,p).

The electrostatic potential (ESP) diagram is a common method to determine the locations with high or low electron density in molecules. It can be used to predict the reaction center of molecules with other materials [30,31]. The electrostatic potential (ESP) diagrams obtained by using the B3LYP/6-31++G(d,p) method are shown in Figure 4. The blue, red, and green region in Figure 4 represents the areas of the most positive, negative, and zero electrostatic potential, respectively. From Figure 4, we found that the highest negative electron density region is located around HSO_4_^−^, while the positive electron density region is mainly located in the imidazolium ring. Therefore, positively charged particles can easily interact with anion HSO_4_^−^, while negatively charged particles can easily interact with the imidazolium ring. The large blue region on the imidazole ring indicates that the imidazole ring would be the main reaction reactive center. In an acidic solution, HSO_4_^−^ could be easily interacted with the positive charge ions (Cu^+^ or Cu^2+^) on the Cu (111) surface, which can reduce the positive charge on the Cu (111) surface.

#### 2.1.5. Reactive Parameters of Three ILs in Solution

Table 2 indicates the quantum chemical parameters of three ILs in solution. From Table 2, we found that the trends of *E*_HOMO_, *E*_LUMO_, Δ*E*, *χ*, *η*, *σ*, Δ*Ν*, and ω are all similar for the results in gas. But the *E*_HOMO_, Δ*Ν* and *ω* in solution are less than that in gas, which suggests that the electron donor role of inhibitors would be decreased by the presence of the solvent The *E*_LUMO_, *χ*, and Δ*E* in solution are higher than in gas, which suggests that the inhibitors have a better tendency to accept electrons from the copper surface in solution and molecular reactivity is decreased by the presence of the solvent. The *µ* and *P* in water solution are greater than in gas, which suggests that the polarization of the entire molecule is stronger in solution. Since the effect of the solvent, the electron acceptor role of imidazolium rings of the corrosion inhibitor has increased, which has a greater tendency to be adsorbed on the copper surface. This result analysis agrees with the results of ESP and the Fukui function.

### 2.2. Molecular Dynamics (MD) Simulation

**Figure 5:**
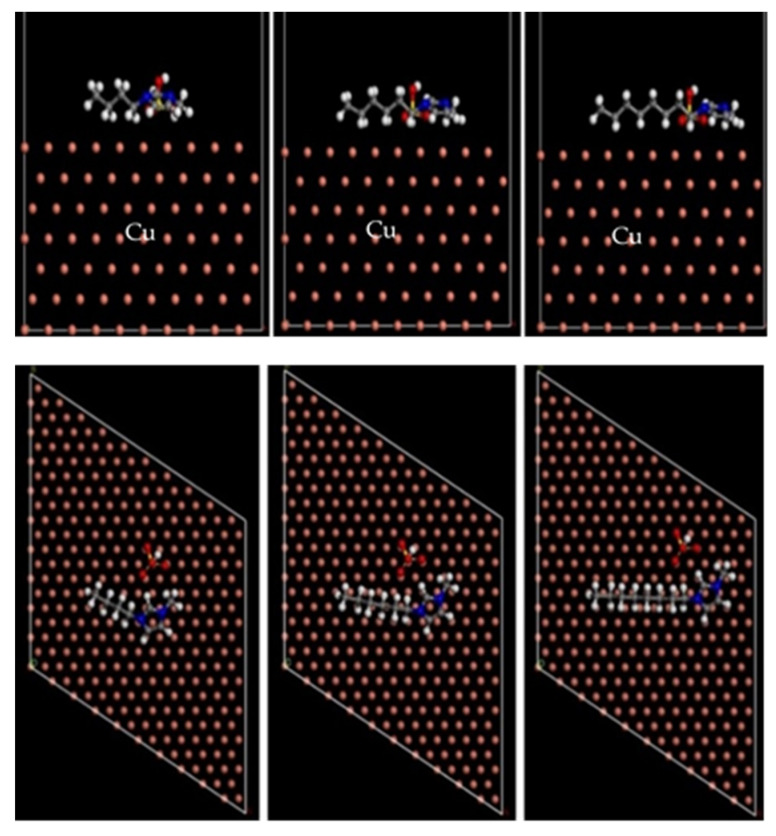
The stable adsorption configurations of [BMIM]HSO_4_, [HMIM]HSO_4_, and [OMIM]HSO_4_ (from left to right) inhibitor on Cu (111) obtained by the MD simulations in a vacuum (**top**: side view, **below**: top view).

**Figure 6:**
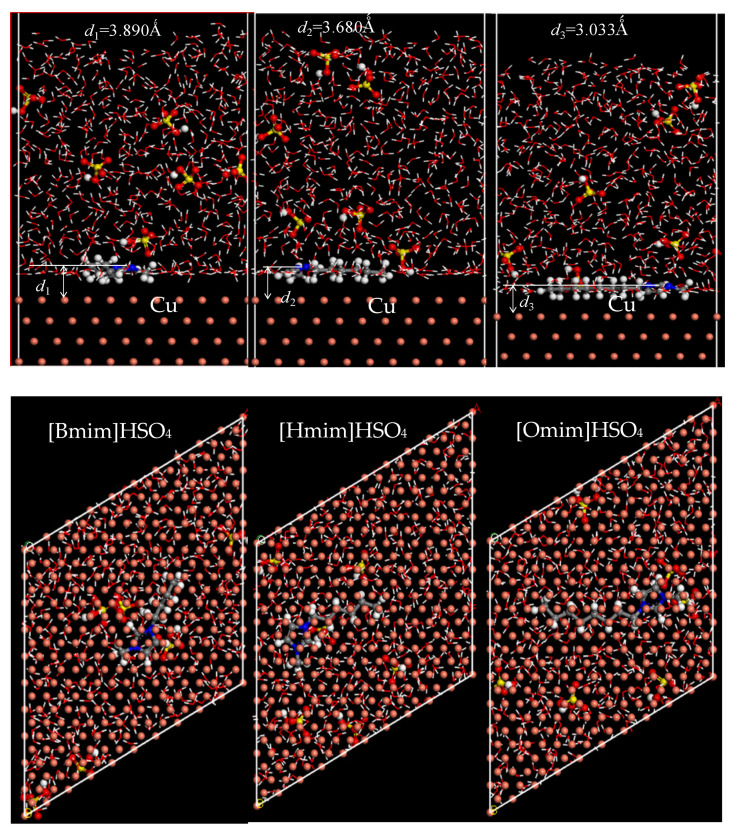
The stable adsorption configurations of [BMIM]HSO_4_, [HMIM]HSO_4_, and [OMIM]HSO_4_ on the Cu(111) surface in sulfuric acid from the MD simulations (**above**: front view, **below**: top view).

The stable equilibrium adsorption configurations of three ILs inhibitors of [BMIM]HSO_4_, [HMIM]HSO_4_, and [OMIM]HSO_4_ on the Cu(111) surfaces in a vacuum and in a sulfuric acid solution, are presented in Figure 5 and Figure 6.

From Figure 5, we can see that the imidazolium ring of the three inhibitors is adsorbed on the Cu(111) surface in parallel. The adsorption energy in a vacuum is presented in Table 3. The larger the absolute value of adsorption energy, the stronger the interaction between the inhibitor molecule and the Cu surface is. As seen in Table 3, the order of the adsorption energy of three ILs inhibitors on the Cu(111) surface in a vacuum is [BMIM]HSO_4_ < [HMIM]HSO_4_ < [OMIM]HSO_4_, which is the same as the order of experimental inhibition efficiencies.

It can be seen from Figure 6 that the imidazole rings of the three ILs inhibitors are adsorbed on the Cu (111) surface in parallel, the distance d between C2 (see Figure 2 for the detail) and the surface is larger than 3 Å, and the distance between other C and N atoms of the imidazole ring, and the copper surface is also greater than 3 Å. The experiment shows that the sum of covalent radii of C and Cu atom is 2 Å [32], and the sum of the covalent radii of the N and Cu atom is 2.03 Å [33], Therefore, the adsorption process between ionic liquid and the copper surface must be dominated by physical adsorption [29,30,31,32,33,34], which is consistent with the standard adsorption free energy analysis obtained by Zhang in the experimental that showed adsorption of the inhibitors on the metal surface is more physical than a chemical one [1]. The order of the distance *d* between the surface and the C2 atom of the three ionic liquids is [Bmim]HSO_4_ > [Hmim]HSO_4_ > [OMIM]HSO_4_. The smaller distance between the C2 atom and copper surface, the stronger interaction between the metal surface and the ILs inhibitor, and the stronger the adsorption of ILs on the Cu(111) surface. Therefore, the interaction between the ILs and Cu surface becomes stronger from [BMIM]HSO_4_ to [OMIM]HSO_4_. As the results of the global reactivity parameters (Δ*E*, *P* and *χ*) in solution, the electrons acceptance ability, and molecular reactivity, become stronger in the order of [BMIM]HSO_4_ < [HMIM]HSO_4_ < [OMIM]HSO_4_ and the effect of the solvent enable the molecule to have more tendencies to accept electrons. With the increase in the alkyl chain, the greater the coverage of molecules on the copper surface is, the higher the corrosion inhibition efficiency is. When the ionic liquid is adsorbed on the copper surface in parallel, the coverage is larger, and the inhibition efficiency is higher.

It is clear from Table 4, that the order of the adsorption energy of three inhibitors on the Cu(111) surface in 0.5 M H_2_SO_4_ solution is [BMIM]HSO_4_ < [HMIM]HSO_4_ < [OMIM]HSO_4_, which means that [OMIM]HSO_4_ is the molecule most easily adsorbed on the surface of Cu (111) to form a protective film, which effectively prevents the adsorption of corrosion ions from the solution to the metal surface, so [OMIM]HSO_4_ is the best corrosion inhibition. Their negative sign indicates a spontaneous interaction of the inhibitor molecule with the corroding copper surface. The results agree well with experimental deduction and observation.

**Figure 7:**
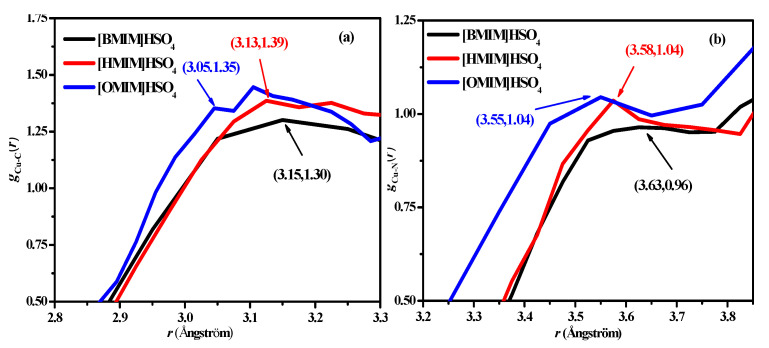
Radial distribution functions of Cu atoms and the C2, N atom in the imidazolium ring of ILs in sulfuric acid from MD simulation. (**a**) RDF *g*_Cu-C_(*r*) of Cu atoms and the C2 atom of ILs, (**b**) RDF *g*_Cu-N_(*r*) of Cu atoms and the N atom of ILs.

Corrosion inhibition mechanisms can be revealed by further analyzing the radial distribution functions *g*(*r*), diffusion coefficient (*D*), and number density profiles [35,36]. The radial distribution function *g*_A-B_(*r*), obtained by molecular dynamics simulation, can be used to analyze the interaction between atom A and atom B. In the sulfuric acid solution, the radial distribution functions *g*_Cu-C_(*r*) and *g*_Cu-N_(*r*) of the Cu surface and the two atoms (C and N) on the imidazole ring of the ILs are shown in Figure 7. It can be seen that the initial peak positions of *g*_Cu-C_(*r*) and *g*_Cu-N_(*r*) for the three ionic liquids of the N and C atoms are [BMIM]HSO_4_ < [Hmim]HSO_4_ < [Omim]HSO_4_, which suggests that the interaction between the Cu(111) surface and the ILs are gradually enhanced from [Bmim]HSO_4_ to [Omim]HSO_4_. The initial peak positions of the radial distribution functions of Cu and C are smaller than those of Cu and N, indicating that the interaction between C and Cu is larger than that between N and Cu. Moreover, the positions of the first peaks of the radial distribution functions of the C and N atoms of the imidazole ring and the copper surface are greater than 3 Å, which is greater than the sum of the covalent bonds between the atoms and the copper surface, indicating a physical adsorption process.

**Figure 8:**
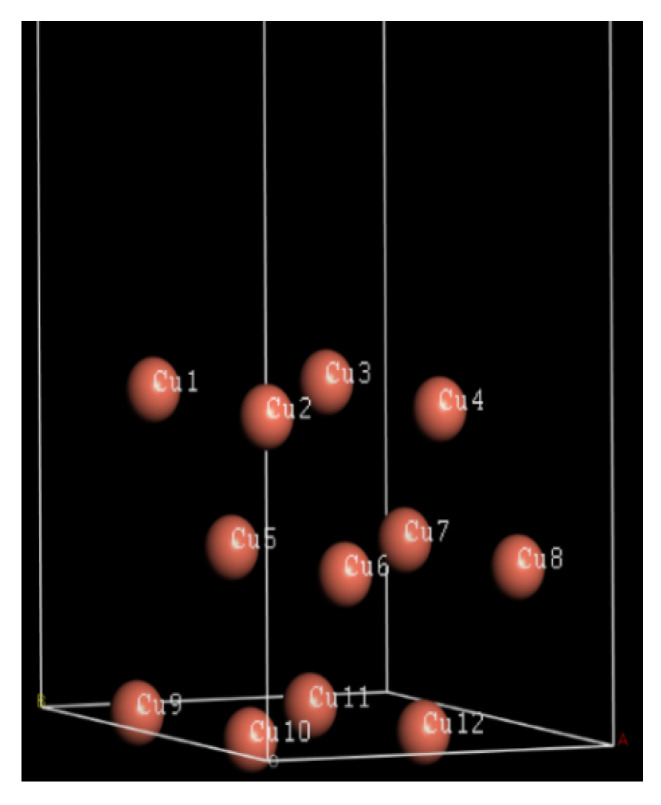
A model of the Cu(111) surface for the quantum chemical calculation with the Castep module of Materials Studio 6.0.

The three-layer copper Cu(111) atoms model is shown in Figure 8. Mulliken charges distribution of Cu(111) atoms in Figure 8 are shown in Table 5. The calculated total Mulliken charges of first layer atoms of the copper surface (Cu1, Cu2, Cu3, and Cu4) are −0.44e. Table 6 shows the Mulliken charge distribution in the imidazolium ring (the main reactive site) and the Cu(111) surface. From Table 6, total charge distribution of imidazolium ring of [BMIM]HSO_4_, [HMIM]HSO_4_, and [OMIM]HSO_4_ are 0.6004e, 0.6376e, and 0.6444e, respectively. The order of the total charge distribution of the imidazolium ring is [BMIM]HSO_4_ < [HMIM]HSO_4_ < [OMIM]HSO_4_. Since the first layer copper atoms are negatively charged, this indicates that the Coulomb interaction between the three ILs and the Cu surface becomes stronger from the order [BMIM]HSO_4_ < [HMIM]HSO_4_ < [OMIM]HSO_4_. This is for the reason that the electrons’ acceptance ability increases from the order [BMIM]HSO_4_ < [HMIM]HSO_4_ < [OMIM]HSO_4_ in Section 2.1.

The diffusion ability and behavior of corrosive particles in the corrosive particles can be described by diffusion coefficient (*D*). *D* can be calculated with the 1/6 of the slope of the mean square displacement (MSD) curve, according to the Einstein diffusive equation [36,37],

(1) $$D=\frac{1}{6}\underset{t\to \infty }{\lim}\frac{d}{dt}\langle {\left[r_{i}\left(t\right)-r_{i}\left(0\right)\right]}^{2}\rangle$$

**Figure 9:**
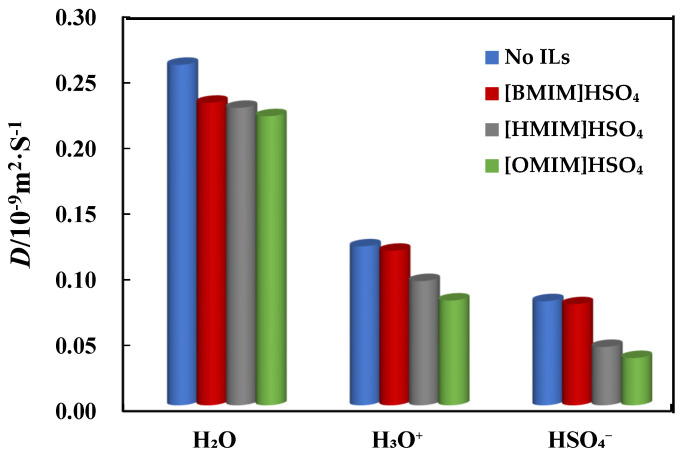
The diffusion coefficient of corrosive particles with addition of three ILs inhibitors from the MD.

In Equation (1), the ${\left[r_{i}\left(t\right)-r_{i}\left(0\right)\right]}^{2}$ is MSD, <…> is the ensemble average, and *N* is the number of particles in the system and *r_i_*(*t*) represents the position vector of the *i* atom or molecule. *D* reflects the diffusion rate of corrosive particles. The diffusion coefficient of three corrosive particles from the molecular dynamics simulation is shown in Figure 9. It can be found that the *D* of the three corrosive particles gradually decreases after adding ILs of [Bmim]HSO_4_, [Hmim]HSO_4_, and [OMIM]HSO_4_, which indicated that the addition of the ILs can effectively inhibit the diffusion of corrosive particles. When ILs are added, they can adsorb onto the Cu(111) surface with the -C=N- of the imidazolium ring and S atom in HSO_4_^−^ to form a protective film, which blocks the transfer of oxygen and corrosive particles from the bulk solution to the copper/solution interface. Therefore, the diffusion coefficient of three corrosive particles gradually decreases with adding ILs. From Figure 9, we found that the order of the inhibition ability is [Bmim]HSO_4_ < [Hmim]HSO_4_ < [OMIM]HSO_4_, and [OMIM]HSO_4_ is the best inhibitor. This is consistent with the above theoretical results (in Section 2.1) and experimental results that the compound [OMIM]HSO_4_ is the best inhibitor and the inhibition ability follows the order: [OMIM]HSO_4_ > [HMIM]HSO_4_ > [BMIM]HSO_4_ [1].

**Figure 10:**
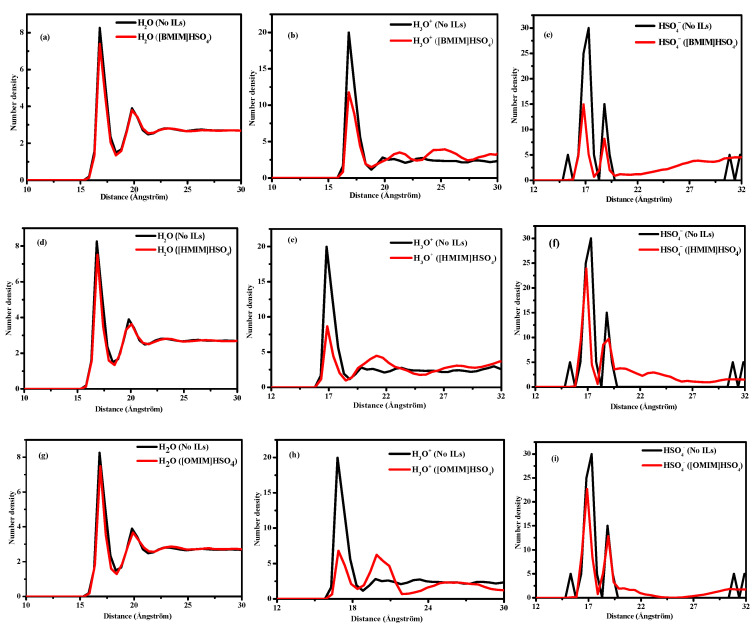
Number density profiles from the molecular dynamics simulations for H_2_O, H_3_O^+^, and HSO_4_^−^ on the Cu(111) surface with and without ionic liquids of [BMIM]HSO_4_ (**a**–**c**), [HMIM]HSO_4_ (**d**–**f**), and [OMIM]HSO_4_ (**g**–**i**).

The number density is used to describe the density distribution of different corrosive particles from one point to the Cu(111) in the vertical direction. Figure 10 displays the number density distribution of the corrosive particles (H_2_O, H_3_O^+^, and HSO_4_^−^) with or without the addition of three ILs molecules.

It can be seen from Figure 10a,c,d that when three ILs are added, the first peak value of the H_2_O molecular number density curve decreases, which means that the addition of inhibitor molecules leads to a decrease in the H_2_O molecular number density on the copper surface. From the number density curves of H_3_O^+^ and HSO_4_^−^, we found that the addition of the ILs inhibitors leads to a significant decrease in the number density of H_3_O^+^ and HSO_4_^−^ on the copper surface. From the discussion in Section 2.1, the ILs are adsorbed on the Cu(111) surface with the -C=N- of the imidazolium ring, O and S atoms in HSO_4_, to form a protective film that blocks the transfer of oxygen and corrosive particles from the bulk solution to the copper/solution interface, which is in good agreement with the experimental deduction in ref [1]. The results show that the longer the branched-chain of the ionic liquid, the stronger the interaction between the ILs and Cu(111) surface, and the closer the adsorption, which hinders the contact between the Cu(111) surface and corrosive particles and greatly slows down the corrosion rate of corrosive ions on the copper surface.

### 2.3. QSAR of Reactive Parameters and the Inhibition Efficiency of Three ILs

The inhibition efficiency (*IE*) of alkylimidazolium ILs was obtained by electrochemical impedance spectroscopy (EIS) measurements in 0.5 M sulfuric acid solution by Zhang et al. [1]. The total inhibition efficiency of three ILs inhibitors in each concentration is correlated with the reactive parameters from the quantum chemical calculation with the linear and nonlinear QSAR models of Equations (8) and (9). The linear results are shown in Equations (2) and (3), while the nonlinear results are given in Equations (4) and (5). It can be seen that the linear regression is not well. For the nonlinear regression, we found that the experimental inhibition efficiencies can be well correlated with the quantum chemical parameter set by the molar mass of molecule *M.wt*, Δ*E*, Δ*N*, and *P*, and the set of *η*, *µ*, Δ*E*, and *P*.

(2) $$IE=0.0012\Delta E+0.0004P-0.0002MV+0.588R^{2}=0.73$$

(3) $$IE=33.741\Delta N-1.9869E+0.2426\mu +0.588R^{2}=0.73$$

(4) $$IE=\frac{\left(-0.014M.wt+0.099\Delta E+59.27\Delta N+0.033P-12.51\right)C_{i}}{1+\left(-0.014M.wt+0.099\Delta E+59.27\Delta N+0.033P-12.51\right)C_{i}}{\;}^{}\;R^{2}=0.99$$

(5) $$IE=\frac{\left(0.203\eta -0.398\mu +0.662\Delta E+0.017P+0.231\right)C_{i}}{1+\left(0.203\eta -0.398\mu +0.662\Delta E+0.017P+0.231\right)C_{i}}R^{2}=0.99$$

**Figure 11:**
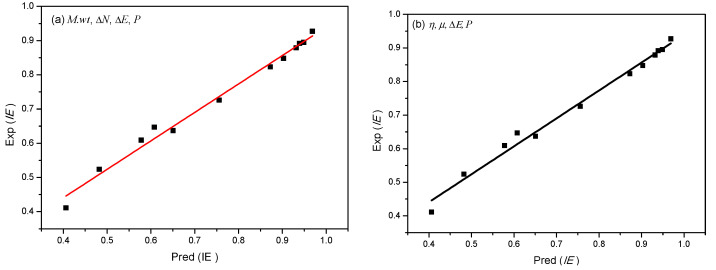
Nonlinear correlation between the predicted inhibition efficiency pred(*IE*) obtained by Equation (4) (**a**) and Equation(5), (**b**) and the experimental value exp(*IE*).

Figure 11 displays the nonlinear correlations between the predicted value pred(*IE*) and experimental values exp(*IE*). It can be seen from Figure 11 that the values of exp(*IE*) and pred(*IE*) are very similar. It is indicated that the established nonlinear correlations of the QSAR models in this study is very reliable. The predicted inhibition efficiency for the three ILs inhibitors by QSAR is [OMIM] HSO_4_ > [HMIM] HSO_4_ > [BMIM]HSO_4_, which agrees well with the experimental results, quantum chemical calculation, and MD simulation.

## 3. Materials and Methods 

### 3.1. Geometry Structure Optimization and the Reactivity of ILs

The geometry optimization and vibrational frequency calculations of three ILs in Figure 1 were performed using Gaussian 09 software [38] with the density functional theory (DFT) method [39]. The B3LYP functional and 6-31++G(d,p) basis set has been used in studying three ionic liquids, which can be compared with the experimental and other theoretical results [40,41]. The vibrational frequency analysis was carried out to ensure that the target molecules have the most stable geometry. All quantum chemical parameters total energy (*E*_total_), the lowest unoccupied molecular orbital energy (*E*_LUMO_), electron affinity (*A* = −*E*_LUMO_), the highest occupied molecular orbital energy (*E*_HOMO_)_,_ ionization potential (***I*** = −*E*_HOMO_), the energy gap (Δ*E* ***= E***_LUMO_ − *E*_HOMO_), dipole moment (*µ*), electrons transferred number (Δ*N*) [42], hardness (*η*), electronegativity (*χ*), softness (*σ*), polarizability (*P*), and electrophilicity (*ω*) [43] were calculated and discussed. The experimental results were obtained in sulfuric acid solution, the effects of the solvent on the molecular reactivity of the three ILs were studied by using the SMD model with the keyword “SCRF = (solvent = water, SMD)” [23] in Gaussian 09.

The local reactivity of a compound can be described by Fukui functions *ƒ_k_*. In a constant external potential, the *ƒ_k_* can be defined as the first-order differential of the electron density *ρ*(*r*) to electron number *N* [44]. The Fukui functions can be given by different approximations as following,

(6) $$f_{k}^{+}=q_{k}^{}(N+1)-q_{k}^{}{\left(N\right)}^{}(\mathrm{for}\;\mathrm{nucleophilic}\;\mathrm{attack})$$

(7) $$f_{k}^{-}=q_{k}^{}(N)-q_{k}^{}{\left(N-1\right)}^{}(\mathrm{for}\;\mathrm{electrophilic}\;\mathrm{attack})$$

where *q_k_*(*N*), *q_k_*(*N*+1), and *q_k_*(*N*−1) are the charges of neutral, cationic, and anionic species, respectively. Here, Fukui indices for the ILs were obtained by Mulliken charges and analyzed by visual graphic surfaces using Multiwfn software [45]. The most susceptible sites to nucleophilic and electrophilic attacks were the regions with the highest values of *ƒ_k_*^+^; and *ƒ_k_*-, respectively.

The inhibition efficiency and quantum chemical descriptors and other molecular indexes can be correlated with the QSAR methods [46]. The linear and nonlinear equations were proposed by Lukocits et al., which are popular and useful to analyze the correlation effects between the corrosion efficiency and quantum chemical parameters. The linear equation Equation (8) [47] and the nonlinear equation Equation (9) are as following [48]

(8) $$IE=Ax_{j}C_{i}+B\;\;\;\;\;$$

(9) $$IE=\frac{\left(Ax_{j}+B\right)C_{i}}{1+\left(Ax_{j}+B\right)C_{i}}$$

where *IE* is the inhibition efficiency, *x_j_* is the quantum chemical parameters of the inhibitor molecule, ***B*** and *A* are regression coefficients, and *C_j_* is the inhibitor concentration.

### 3.2. Adsorption of ILs on Copper Surface and the Inhibition Mechanism

Molecular dynamics simulation (MD) has been successfully and widely applied to study the inhibition behavior and mechanism of inhibitors on metal surfaces [49,50]. The adsorption progress of three alkylimidazolium ionic liquids on the Cu (111) surface was examined by molecular dynamics simulation using the Forcite module in Materials Studio [51]. Cu(111) surfaces were chosen for the simulation because they are a more stable and denser surface [32,33,34], which have been widely used to study the adsorption of copper surfaces with other organic inhibitors [1,2,3,4,5,6,7,8,9,10,11,12]. The simulation box includes a surface, a material layer, and a vacuum from bottom to top. The Cu(111) surface had seven layers of copper atoms and the Cu(111) plane was enlarged to 10 × 10 supercells. For the vacuum simulation, the material layer contained one IL inhibitor molecule. The vacuum slab was 30 Å. To have an experimental concentration of 0.5 M H_2_SO_4_, 495 H_2_O molecules, 5 ions of H_3_O^+^, 5 ions of HSO_4_^−^, and 1 ILs were placed in the material layer of the simulation box. The MD simulation was conducted in NVT canonical ensemble using an Anderson thermostat at 303 K. The total simulation time was 1000 ps with a time step of 1.0 fs. The COMPASS II force fields were utilized to optimize the structures of all components of the system [52]. The cutoff radius was 15.5 Å. The trajectory was recorded every 100 steps for subsequent analysis.

The adsorption energy between the Cu(111) surface and the inhibitors in the vacuum medium is given as follows [53]

(10) $$E_{\mathrm{adsorption}}=E_{\mathrm{total}}{-}^{}\left(E_{\mathrm{surface}}{+}^{}E_{\mathrm{inhibitor}}\right)$$

where *E*_surface_ is the energy of the surface *E*_inhibitor_ is the energy of the inhibitor molecule adsorbed on the surface and *E*_adsorption_ is the adsorption energy.

The adsorption energy between the Cu(111) surface and the ILs inhibitors in the acidic medium is given as following [24],

(11) $$E_{\mathrm{adsorption}}=E_{\mathrm{total}}{-}^{}\left(E_{\mathrm{surface}+\mathrm{solution}}{+}^{}E_{\mathrm{inhibitor}+\mathrm{solution}}\right)+E_{\mathrm{solution}}\;$$

where *E*_adsorption_ is the adsorption energy, *E*_onhibitor+solution_ is the energy of the sulfuric acid solution and inhibitor molecule, *E*_surface+solution_ is the energy of the sulfuric acid solution and surface, *E*_solution_ is the energy of the sulfuric acid solution.

The radial distribution function and number density curve was analyzed to obtain some adsorption properties. Corrosion media particles, including H_2_O, H_3_O^+^, and HSO_4_^−^ would diffuse to the metal surface. The effect of corrosion inhibitors on the diffusion behavior of particles on the metal surface can be obtained by analyzing the self-diffusion coefficient (*D*) and the number density curve. The radial distribution function curves of C and N can be utilized to analyze the interaction between molecules and the Cu(111) surface.

The charge difference of the surface before and after adsorption was calculated by the CASTEP module of Materials Studio software. The GGA-PW91 functional with ultra-soft pseudo-potentials, an energy cut-off of 350 eV, and a k-point of 2 × 2 × 1 were used in geometry optimization and property analysis. The Cu(111) surface was enlarged to a (2 × 2) super cell with 3-layer copper atoms. A vacuum slab of 10 Å was placed above the Cu(111) surface.

## 4. Conclusions

Global reactivity parameters reveal that the variety of these parameters are with the difference of electrons transferred ability and molecular reactivity, and the adsorption ability from low to high is [BMIM]HSO_4_ < [HMIM]HSO_4_ < [OMIM]HSO_4_. HOMO, LUMO distribution, ESP, and Fukui functions *ƒ_k_* suggest that the main reactive site is the imidazolium ring (especially the C2, N4, and N7 groups). The increase in chain length of the imidazolium ring increases the total charge on the imidazolium ring and the distribution of HOMO, LUMO*, **ƒ***^+^, and *ƒ*^−^, making it easier for ILs to give or accept electrons. Quantum chemical calculations predict that the orders of the inhibition efficiency in gas and solution are both [BMIM]HSO_4_ < [HMIM]HSO_4_ < [OMIM]HSO_4_, which are consistent with the experimental results.

Molecular dynamics simulation results show that the imidazole ring of ILs tends to be adsorbed on the Cu(111) surface in parallel through physical adsorption. The order of adsorption energy is [Bmim]HSO_4_ < [Hmim]HSO_4_ < [OMIM]HSO_4_, which agrees well with the experimental order of corrosion efficiency. On the imidazole ring, the interaction between the copper surface and the C atom is greater than that between the copper surface and the N atom. The diffusion coefficient of H_2_O, H_3_O^+^, and HSO_4_^−^ is decreased when ILs are added. This reflects that the three ILs have a strong ability to restrict the diffusion of these corrosive particles, and the corrosion resistance increases from [BMIM]HSO_4_ to [OMIM]HSO_4_; these observations are consistent with previous results. The number density curve of corrosive particles suggests that three ILs can drive away H_2_O molecules, H_3_O^+^, and HSO_4_^−^ from the Cu surface effectively.

A good QSAR correlation between the corrosion inhibition efficiency can be correlated with the quantum chemical parameters of the studied ILs, and the theoretical predictions agree well with the experimental results.

## Figures and Tables

**Table 1:** Fourteen reactive parameters of three ILs in the gas phase obtained by B3LYP/6-31++G(d,p).

	[BMIM]HSO_4_	[HMIM]HSO_4_	[OMIM]HSO_4_
*E*_total_ (au)	−1123.1	−1201.7	−1280.3
*E*_HOMO_ (ev)	−6.3138	−6.3064	−6.2289
*E*_LUMO_ (ev)	−1.3271	−1.3260	−1.2664
Δ*E* (ev)	4.9867	4.9804	4.9625
μ	12.814	12.620	10.905
*P*	144.54	169.69	193.14
*χ* (ev)	3.8205	3.8162	3.7477
*η* (ev)	2.4934	2.4902	2.4813
*σ* (ev)	0.4011	0.4016	0.4030
Δ*Ν*	0.2024	0.2036	0.2181
*Ι* (ev)	1.3271	1.3260	1.2664
*A* (ev)	6.3138	6.3064	6.2289
*ω* (ev)	2.9270	2.9241	2.8302
*MV* (cm^3^/mol)	161.45	199.07	217.35

**Table 2:** Reactive parameters of three ILs in solution obtained with 6-31++G(d,p).

	[BMIM]HSO_4_	[HMIM]HSO_4_	[OMIM]HSO_4_
*E*_total_ (au)	−1123.1	−1201.8	−1280.4
*E*_HOMO_ (ev)	−7.3692	−7.3527	−7.3236
*E*_LUMO_ (ev)	−0.6825	−0.6669	−0.6457
Δ*E* (ev)	6.6867	6.6858	6.6779
*µ* (D)	27.236	23.473	22.116
*χ* (ev)	4.0259	4.0098	3.9847
*η* (ev)	3.3434	3.3429	3.3390
*σ* (ev)	0.2991	0.2991	0.2995
Δ*Ν*	0.4448	0.4472	0.4515
*Ι* (ev)	7.3603	7.3527	7.3236
*A* (ev)	0.6789	0.6669	0.6457
*ω* (ev)	2.4238	2.4049	2.3776
*P*	191.26	225.373	260.28

**Table 3:** Adsorption energies (kcal/mol) of inhibitors on the Cu(111) surface from an MD simulation in a vacuum.

ILs	[BMIM]HSO_4_	[HMIM]HSO_4_	[OMIM]HSO_4_
*E*_adsorptio_	−58.737	−69.866	−81.471

**Table 4:** Adsorption energies of three ILs inhibitors in sulfuric acid solution from an MD simulation (kcal/mol).

ILs	[BMIM]HSO_4_	[HMIM]HSO_4_	[OMIM]HSO_4_
*E*_adsorption_	−59.080	−71.154	−83.134

**Table 5:** Mulliken charges of the Cu(111) surface.

Atom	Number	Charge
Cu	1	−0.11
Cu	2	−0.11
Cu	3	−0.11
Cu	4	−0.11
Cu	5	0.22
Cu	6	0.22
Cu	7	0.22
Cu	8	0.22
Cu	9	−0.11
Cu	10	−0.11
Cu	11	−0.11
Cu	12	−0.11

**Table 6:** Mulliken charges of atoms in the imidazolium ring.

Atom	[BMIM]HSO_4_	[HMIM]HSO_4_	[OMIM]HSO_4_
C2	−0.6686	−0.5758	−0.8694
C1	0.0184	0.0032	0.0701
C3	−0.0320	−0.0225	0.1357
N4	−0.0331	−0.0216	0.2111
H5	0.1643	0.1629	0.1722
H6	0.1476	0.1453	0.1593
N7	0.0631	0.0570	0.1440
C14	−0.2246	−0.2202	−0.2341
H15 H16	0.2363 0.1817	0.2328 0.1820	0.2298 0.1793
H17	0.1833	0.1851	0.1964
H21	0.5641	0.5094	0.2502

## Data Availability

Data is contained within the article.
